# Nanomechanical Stability of Aβ Tetramers and Fibril-like Structures: Molecular Dynamics Simulations

**DOI:** 10.1021/acs.jpcb.1c02322

**Published:** 2021-07-12

**Authors:** Adolfo B. Poma, Tran Thi Minh Thu, Lam Tang Minh Tri, Hoang Linh Nguyen, Mai Suan Li

**Affiliations:** † Institute of Fundamental Technological Research, Polish Academy of Sciences, Pawińskiego 5B, 02-106 Warsaw, Poland; ‡ Institute for Computational Science and Technology, SBI Building, Quang Trung Software City, Tan Chanh Hiep Ward, District 12, Ho Chi Minh City, Vietnam; § Faculty of Materials Science and Technology, Ho Chi Minh City University of Science - VNUHCM, 227 Nguyen Van Cu Street, District 5, Ho Chi Minh City, Vietnam; ∥ Ho Chi Minh City University of Technology (HCMUT), Ho Chi Minh City 700000, Vietnam; ⊥ Vietnam National University, Ho Chi Minh City 700000, Vietnam; # Institute of Physics, Polish Academy of Sciences, Al. Lotników 32/46, 02-668 Warsaw, Poland; % International Center for Research on Innovative Biobased Materials (ICRI-BioM)—International Research Agenda, Lodz University of Technology, Żeromskiego 116, 90-924 Lodz, Poland

## Abstract

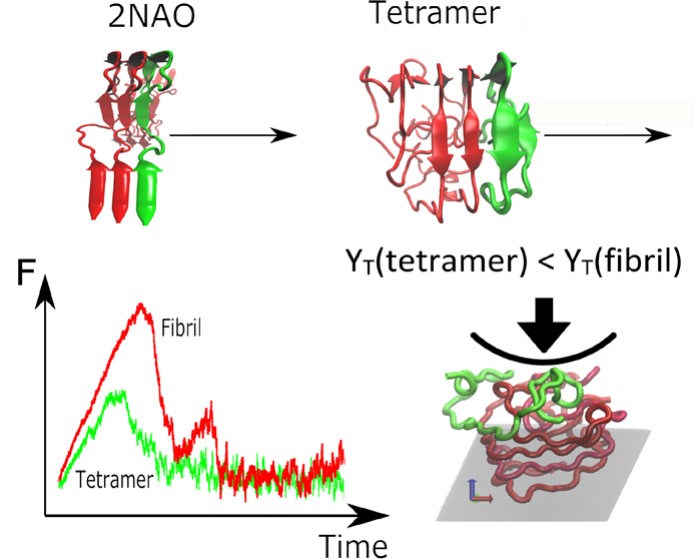

Alzheimer’s disease (AD) is a neurodegenerative disorder and one of the main causes of dementia. The disease is associated with amyloid beta (Aβ) peptide aggregation forming initial clusters and then fibril structure and plaques. Other neurodegenerative diseases such as type 2 diabetes, amyotrophic lateral sclerosis, and Parkinson’s disease follow a similar mechanism. Therefore, inhibition of Aβ aggregation is considered an effective way to prevent AD. Recent experiments have provided evidence that oligomers are more toxic agents than mature fibrils, prompting researchers to investigate various factors that may influence their properties. One of these factors is nanomechanical stability, which plays an important role in the self-assembly of Aβ and possibly other proteins. This stability is also likely to be related to cell toxicity. In this work, we compare the mechanical stability of Aβ-tetramers and fibrillar structures using a structure-based coarse-grained (CG) approach and all-atom molecular dynamics simulation. Our results support the evidence for an increase in mechanical stability during the Aβ fibrillization process, which is consistent with *in vitro* AFM characterization of Aβ_42_ oligomers. Namely, using a CG model, we showed that the Young modulus of tetramers is lower than that of fibrils and, as follows from the experiment, is about 1 GPa. Hydrogen bonds are the dominant contribution to the detachment of one chain from the Aβ fibril fragment. They tend to be more organized along the pulling direction, whereas in the Aβ tetramers no preference is observed.

## Introduction

According to the amyloid cascade hypothesis,^1^ Alzheimer’s disease (AD) is caused by extracellular aggregation of amyloid beta (Aβ) peptides, leading to the formation of fibrils and plaques. This hypothesis may be true for other diseases such as Parkinson’s disease, type II diabetes, amyotrophic lateral sclerosis, and so on, but with the accumulation of other proteins.^2^ Initially, mature Aβ fibrils with a cross-β-sheet structure were considered to be neurotoxic argents, but later experiments showed that soluble oligomers of the 2–32 chains are more toxic.^3−5^ Aβ_42_ (peptide of 42 amino acids), but not Aβ_40_ (40 amino acids), oligomers have been found to form pores in lipid membranes, resulting in a loss of ionic homeostasis.^6,7^ This result is consistent with the observation that Aβ_42_ tetramer or larger oligomers transversal to the neuron membrane and calcium ions enter the cell, causing the neurotoxicity.^8^

Recently, Ruggeri et al.^9^ demonstrated the first *in vitro* experimental evidence of a difference in nanomechanical stability between the Aβ clusters, protofibrils, and extended fibril-like structures in Aβ_42_ systems. The range of the Young modulus reported varies between 1 and 3 GPa for Aβ clusters and mature fibrils.

In this study we shed light onto the mechanical stability of the Aβ clusters and ordered fibril-like structures under nonequilibrium forces. If we consider protein–protein interactions, for example, we know that the amount of mechanical force that a protein complex can resist before breakage can be decorrelated from its binding affinity which is dominated by thermodynamics. By use of biophysical tools such as the atomic force microscope (AFM), it is possible to study *in vitro* the mechanical responses of small molecular complexes that, when mechanically stressed, dissociate along energetic pathways that are inaccessible under purely thermal excitation. These properties lead to diverse mechano-responsive behaviors in biological systems, such as force-activated catch bonds,^10^ mechano-chemistry response in GPCR molecules,^11^ and enhanced cell adhesion of pathogens under Brownian motion.^12^ Typical nanoindentation is based on AFM in contact mode which allows to quantify the resistance of individual biomolecules (e.g., protein, polysaccharide, and nucleic acids) and their molecular complexes under loading forces in the range of piconewtons–nanonewtons. The aim is to understand how mechanical stability plays a role in adhesion of proteins and how they perform their intended functions at the molecular level. Through these experiments one can understand what makes protein interactions mechanically strong and characterize the system via the determination of the elastic constants (i.e., Young modulus).

Molecular simulation offers a reliable, reusable, and cost-effective way of investigating the mechanical stability of individual protein chains and protein complexes; furthermore, the molecular mechanism that triggers those responses can be elucidated. One common technique that characterizes the nanomechanics of protein complexes is steered molecular dynamics (SMD) simulation.^13,14^ The success of this approach comes from the characterization of a series of events needed to unfold a single or a protein complex at the atomistic resolution. The SMD can reproduce various structural characteristics of the unfolding events, and it has been used for the study of tensile deformation in β-amyloid fibrils,^15,16^ elucidating the mechanism of high stability in the nanonewtons range for cohesin–dockerin binding,^17^ and so on. In addition, a structure-based coarse-grained (CG) model based on one atom (C_α_) per amino acid is more suitable to capture an essential picture of deformation for a very large system and longer time scales.^18^ They remove several degrees of freedom of the system, which enables one to reach the experimental time and length scales required to describe the relevant phenomena, while maintaining the description of the system under consideration at the molecular level. In particular, our CG model can be used to infer the elastic parameters in ideal conditions (solvent free). When it actually follows a theoretical model, the elastic modulus can be obtained from the linear response. Most importantly, the mechanism of deformation that gives rise to the linear response can be characterized in a CG simulation.

In this study we combined all-atom MD and structure-based CG to quantify the mechanical stability of the Aβ tetramers through SMD simulation and CG nanoindentation. Our results agree with AFM experiments^9^ and provide a quantitative description of the mechanical gain during the Aβ fibril growth.

## Materials and Methods

### Steered Molecular Dynamics (SMD) Simulation

The atomic structure of Aβ fibrils was obtained by various experimental techniques, including solid-state NMR and cryo-EM. However, the structure of small oligomers cannot be resolved experimentally due to their transient nature that comes from fast aggregation in solution. In this situation MD simulations were used to obtain their structures.^2,19−21^

**Figure 1:**
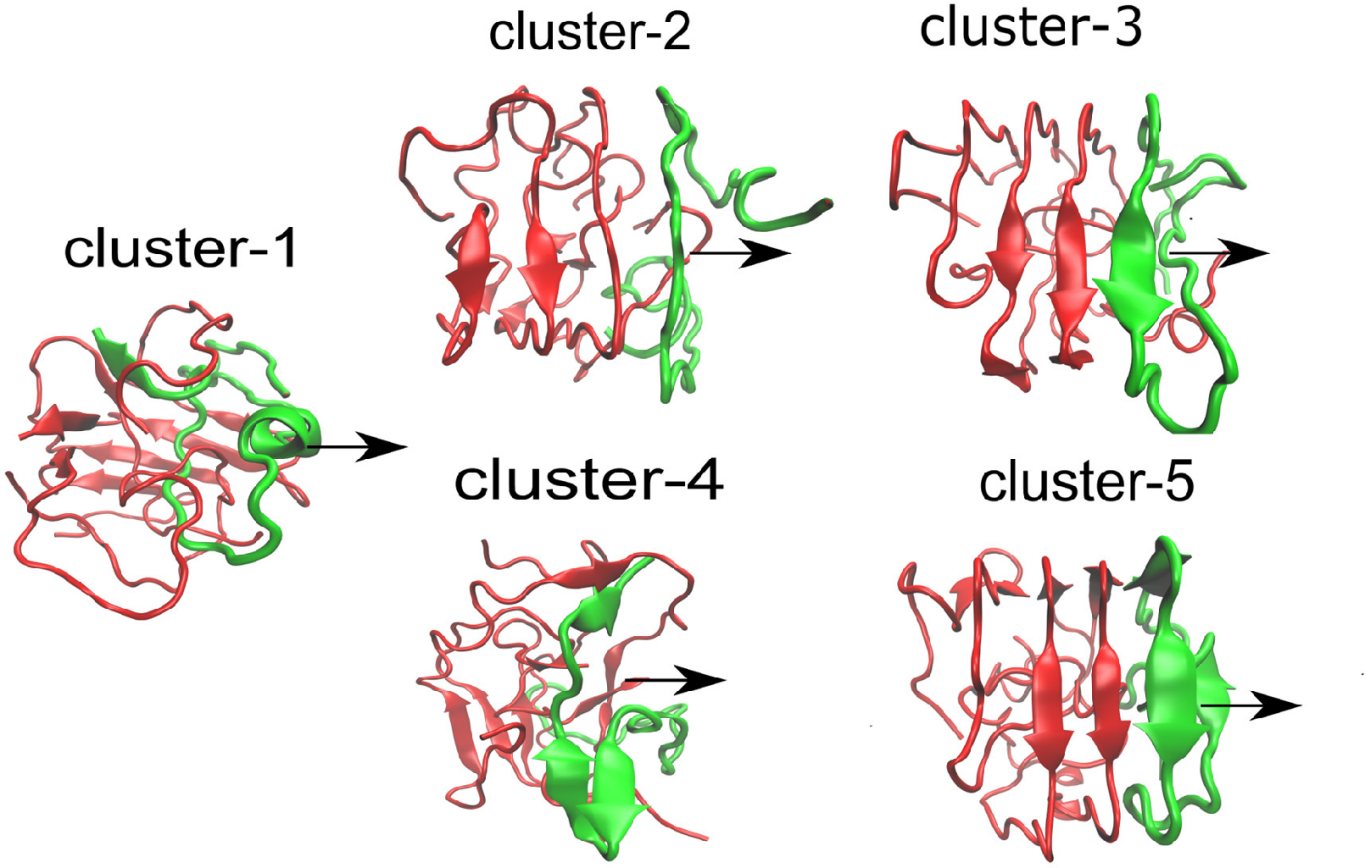
Computational models of Aβ tetramers taken from Nguyen et al.^19^ The arrows indicate the pulling direction in SMD simulation.

**Figure 2:**
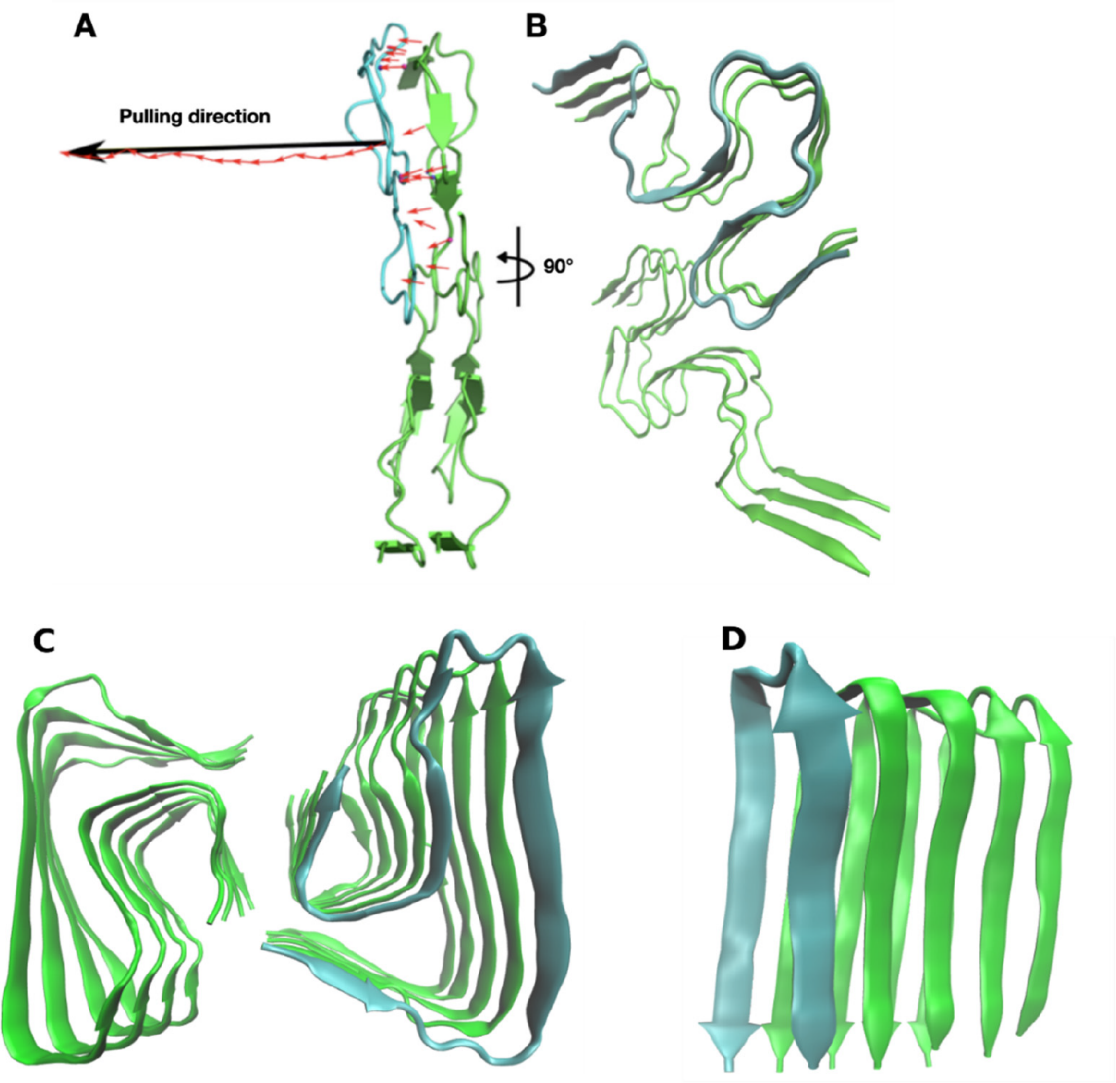
Aβ fibril fragment (PDB code 2NAO) has a 2-fold symmetry. Panel A shows the vector of interchain HBs at the interface (red) and the total vector (black). The pulling direction was chosen along this total vector. Panel B depicts the Aβ system rotated by 90°. Panels C and D represent the fibril structures with PDB code 5OQV and 2BEG, respectively.

Here we used the initial structures of the Aβ tetramers (see Figure 1), which were obtained from our previously reported study^19^ by all-atom MD simulation with the OPLS-AA/L force field in explicit solvent with the TIP3P water model. The structures of Aβ_42_ protofibrils were retrieved from Protein Data Bank (PDB) with code 2NAO(22) (Figure 2A,B), 5OQV(23) (Figure 2C), and 2BEG(24) (Figure 2D). Because our simulation will be done at pH 7, but 50QV was obtained at pH 2, we must check its stability at pH 7. As can be seen from Figure S1 in the Supporting Information, the root-mean-square deviation (RMSD) of Cα atoms remains below 0.4 nm, which indicates that 5OQV is also stable at pH 7 and can be used in our simulations.

To generate starting structures for SMD simulations, at the first stage of simulation, all systems were minimized by the steepest-descent method and equilibrated under a constant volume (NVT) ensemble in 1 ns and constant pressure (NPT) in 1 ns, maintaining an isotropic pressure at 1.0 bar. Initial random velocities were generated from the Maxwell distribution at 300 K. We used the *v*-rescale thermostat to keep the temperature close to 300 K.^25^ The pressure was kept fixed by the Parrinello–Rahman algorithms.^26^ A physiological salt concentration of 150 mM was used. The last structure obtained in the equilibration step was used as starting conformation for SMD simulations.

The choice of pulling direction in SMD simulation of a protein–protein complex is not unique, and one of the possible choices was described in Nguyen et al.^27^ On the basis of the fact that hydrogen bonds (HBs) between two subunits play a key role in the stability of the complex, we choose the pulling pathway in the direction of the total HB vector (see Figure 2A). In fact, this direction of pulling maximizes the response (HB breaking) of the system under load (a HB is considered to be present when the distance between donor atom and acceptor atom is less than 0.35 nm and the angle between acceptor-H-donor atom is larger than 135°). HB between two monomers was represented as a vector by using Pymol software. Each HB has two possible directions, and we choose the direction that maximizes the sum of all vectors (see Figure 2A). The pulling direction, obtained by our protocol for the tetramer models, is shown in Figure 1.

Proteins were placed in a rectangular box that is large enough to have space for pulling simulations and satisfy the minimum image convention condition. For Aβ tetramers, except the chain to which the external force was applied, all the remaining chains were restrained to prevent them from drifting due to pulling. Unlike tetramers, in the case of protofibrils, only the neighbor chain was used as an immobile reference for the pulling simulations. An external force was applied to one end of a spring that is attached to the center of mass (COM) of one of the monomers and pulled it along the arrow shown in Figures 1 and 2.

The spring constant (*k*) of a spring connecting a dummy atom and COM of the pulled chain was set equal to 239 kcal/(mol nm^2^) (or 1000 kJ/(mol nm^2^)) as in AFM experiments.^27,28^ Because of limited computational facilities, the pulling speed (*v*) in SMD simulation was chosen to be 1 nm/ns, which is much larger than its experimental reference value, but the qualitative results should not depend on *v*. The force experienced by the pulled peptide was calculated as *F = k*(*vt – x*), where *x* denotes its displacement from the initial position. If the interactions that stabilize the fibril structure are disrupted, the single chain detaches from the core of the performed template, resulting in a force drop as resistance disappears. Thus, similar to the stretching of a single protein chain, *F*_max_ can characterize the mechanical stability of the entire fibril. To obtain a force–extension profile, for each time step, the resulting force was computed. For each case, we performed five trajectories and the maximum force and pulling work were calculated as the average value. From the force–time profile, we collected the rupture force *F*_max_, a force needed for the protein–protein dissociation. Moreover, Vuong et al.^29^ showed that the pulling work *W*_pull_ better describes experiment than the rupture force because it is a function of the entire process, while *F*_max_ is determined only in a single state. Thus, we can use the pulling work as a score function for measuring the mechanical stability of the fibril. The pulling work is computed by the following equation:
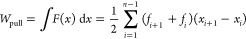
1All systems were simulated with the TIP3P water model and CHARMM36m force field,^30^ which was developed for intrinsically disordered proteins such as Aβ peptides. Indeed, this force field has been validated in previous studies of Aβ_42_ monomers^19^ and oligomers.^20^ The GROMACS package, ver. 5.1.2,^31^ was the molecular dynamics engine.

## Nanomechanics of Proteins: A Coarse-Grained Model

We have employed the structure-based CG approach (i.e., Go̅-like model) that has been tuned by all-atom MD and experimental rupture forces.^18,32^ Our approach is based on a well-determined structure which serves to devise a contact map (CM) of native interactions. In general, one can take a deposited protein structure from the Protein Data Bank to obtain a CM or use MD simulation to construct a dynamical CM.^33^ Our approach has been validated in several applications from the reconstruction of protein sequences,^34^ stretching of biomolecules, and modeling of large conformational changes in protein complexes.^35−37^ This methodology has been successful to map those changes in protein assemblies under applied large forces inducing local deformations such as during nanoindentation. Thus, we performed a computational nanoindentation study which has been validated for single transmembrane proteins and biological filaments.^18,38,39^ In the case of nanomechanical indentation we consider a spherical object with the radius of curvature *R*_ind_ that is in contact with the amyloid cluster and press against the direction of maximal hydrogen bond contribution with a speed of *v*_ind_. Once the indenter travels inside the cluster the distance of *h* away from the undeformed situation, then the force of reaction generated by the cluster is *F*(*h*). This process is continued until the indenter reaches a penetration depth which typically is about 0.5–1 nm. The relationship that holds *F* and *h* is given by Hertz elastic model,^40^ which is equal to , where ν denotes the Poisson ratio of the protein assembly. It is defined as the ratio of the transverse contraction strain to the axial strain in the direction of the stretching deformation. In this case we took it equal to 0.5.

The linear part of the *F* vs *h*^3/2^ profile provides a direct calculation of the Young modulus (*Y*_T_). In experiments, the indenter has a finite stiffness, and the resulting compliance has to be subtracted from the full indentation depth. In simulations, we can eliminate the compliance of the indenter by making it sufficiently stiff. It should be commented that during the indentation process we went further than the reversible region, and we carry out several indentation tests (*n* = 100) to account for the standard deviation (SD) in the process.

## Results and Discussion

### All-Atom Steered Molecular Dynamics Simulation

Previous studies proved that the maximum force *F*_max_ can be used as a measure of the mechanical stability of proteins.^41−43^ This concept has also been useful in identifying a possible relationship between mechanical stability and protein aggregation rate that the higher the aggregation rate, the more stable the fibrillar structure.^44^ This hypothesis is supported by the fact that Aβ_42_ aggregates faster than Aβ_40_^45,46^ because the rupture force of Aβ_42_ fibril is higher than Aβ_40_.^44^ Here both *F*_max_ and *W*_pull_ will be used to characterize the nanostability of oligomers and mature fibrils. It is also interesting to note that Chakraborty et al.^47^ have shown that the difference in the rate of aggregation of Aβ_42_ and Aβ_40_ is associated with a higher population of the so-called fibril-prone state N* (N* is defined as the conformation of a monomer in a fibril state) of Aβ_42_ compared to Aβ_40_. This is consistent with the theory developed by Li et al.^48,49^ showing that the fibril formation time is controlled by the population of N*.

As mentioned above, Ruggeri et al.^9^ reported that Aβ_42_ fibrils are more stable than Aβ_42_ oligomers. To check this experimental result, we first performed SMD simulation for the Aβ_42_ tetramer with five representative models (Figure 1) and two fibril models 2NAO and 5OQV. To shed light on the effect of fibril morphology on mechanical stability, we also examined the 2BEG model^24^ of the truncated peptide Aβ_17–42_.

### Mechanical Unfolding Pathways of the Tetramer Are More Complex than the Fibril

**Figure 3:**
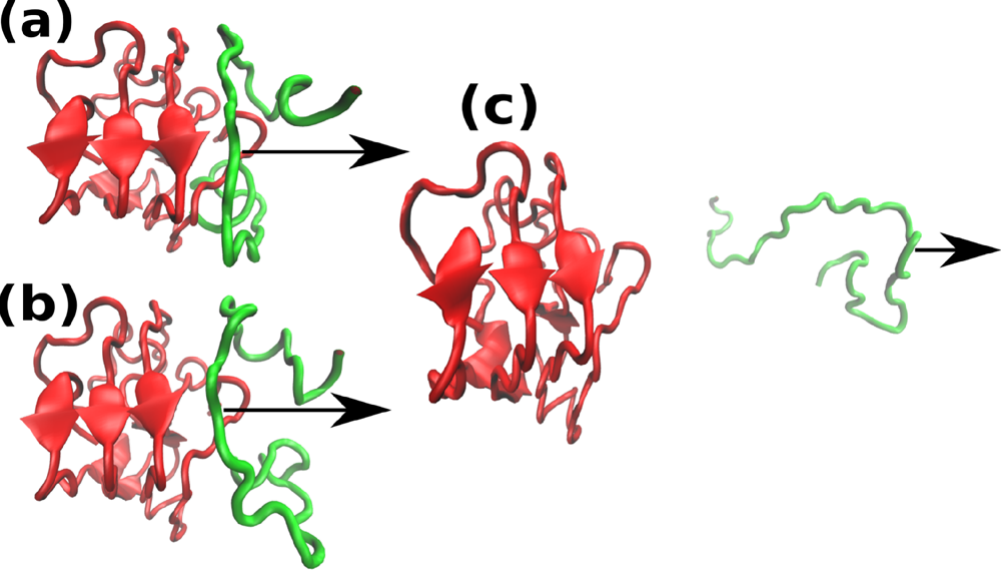
Snapshots of the Aβ tetramer at different displacement between COM of two chain along the pulling path (*x*-axis): (a) *x* = 1.30 nm (initial state, *t* = 0), (b) *x* = 1.75 nm (at the maximum force, *t* = 790 ps), and (c) *x* = 5.8 nm (the pulled chain is completely separated, *t* = 4500 ps).

**Figure 4:**
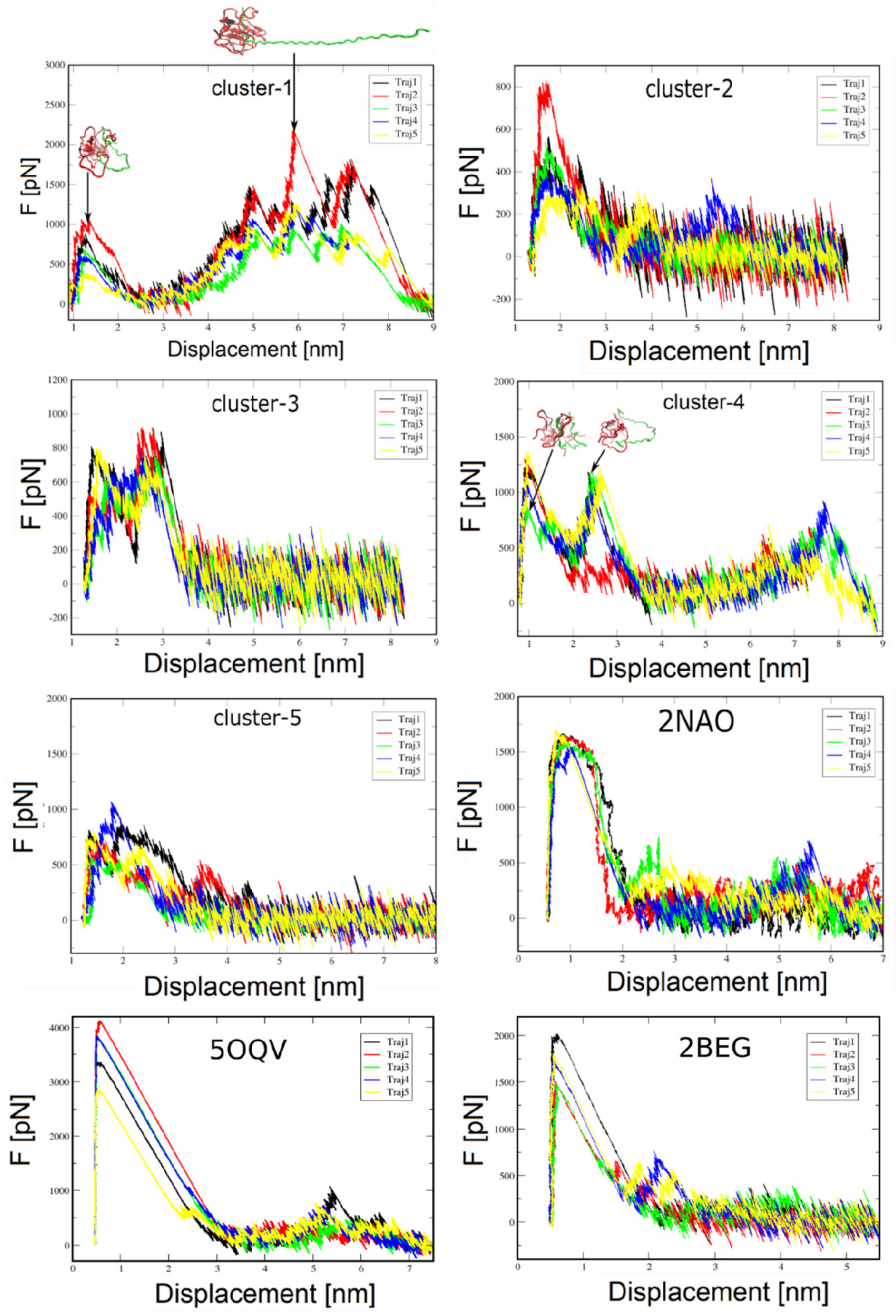
Force–displacement profile of SMD simulations. A chain is pulled out of the Aβ fibril fragment. *F*_max_ measures mechanical stability. For Aβ tetramer clusters namely 1 and 4 are shown the structure of the chain at two instances of the pulling simulation. At the bottom we show the results for three fibrils 2NAO, 5OQV, and 2BEG.

Figure 3 shows the snapshots of the Aβ_42_ tetramer during the pulling process and the time dependence of the pulling work in one SMD trajectory. Clearly, the oligomer remains compact after detachment of one chain, which may be associated with rapid pulling. In the case of the tetramer, the force exerted by a pulled chain versus displacement exhibits complex behavior depending on computational models (Figure 4). In particular, for clusters 1 and 4, several peaks are observed due to the entanglement of chains arising during unfolding. In trajectory 1 of model 1 (Figure 4) the pulled chain is still compact at the first peak. The system spends a lot of time on the second peak, when the pulled chain nearly unfolds because it is clinging to another chain. This clinging itself resulted in a great rupture force.

Because the chains in the initial (PDB) structures 2NAO, 5OQV, and 2BEG are well separated, there is only one peak in the force–displacement profile (Figure 4), which implies that unfolding pathways of the mature fibril are less diverse than the tetramer. To better understand this problem, we built a contact map that shows only those “native” contacts that existed in the initial configuration but are broken at *F*_max_ (Figure S2). Because the “native” contacts of the tetramer disappear in more different positions than the fibril, the detachment pathways in the first case are more complex than in the second. The number of hydrophobic–hydrophobic, hydrophobic–hydrophilic, and hydrophilic–hydrophilic contacts at the initial moment of time and rupture is indicated in Table S1, which suggests that the hydrophobic–hydrophilic interactions are less important than others.

### Aβ Tetramers Are Mechanically Less Stable than Fibril-like Structures

The rupture forces obtained in all trajectories are reported in Table S2, and averaging over 20 runs we have *F*_max_ = 2065, 703, 817, 1226, and 981 pN for clusters 1, 2, 3, 4, and 5, respectively. Averaging over five clusters gives *F*_max_ = 1158 pN for the tetramer (Table 1). The average rupture force is 1534 and 4200 pN for 2NAO and 5OQV, respectively (Table 1), which is higher than that of the tetramer (1158 pN). Therefore, in agreement with Ruggeri et al.,^9^ mature fibrils are mechanically more stable than oligomers.

**Table 1:** Average Rupture Force *F*_max_ and Pulling Work *W*_pull_ of Aβ Tetramer and Fibril-like Structures^a^

systems	*F*_max_ (pN)	*W*_pull_ (kcal/mol)
tetramer	1158 ± 169	347 ± 51
2NAO	1534 ± 166	312 ± 29
5OQV	4200 ± 560	977 ± 169
2BEG	1416 ± 219	188 ± 42

a The results were obtained by using 20 SMD trajectories and pulling speed *v* = 1 nm/ns.

**Figure 5:**
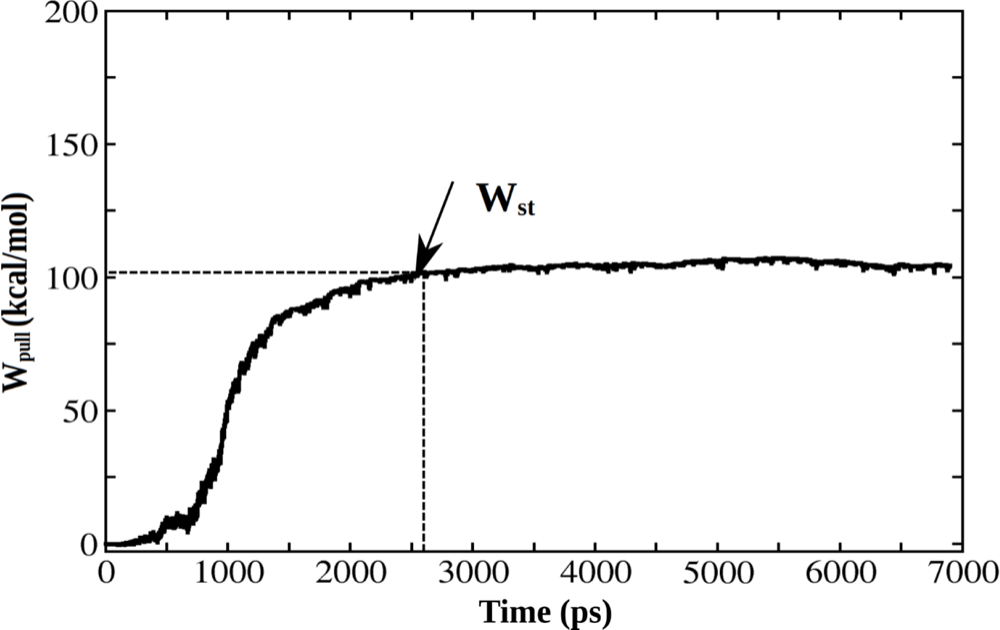
Time dependence of the pulling work with *W*_st_ at the saturation point.

Because there is no interaction between the pulled chain and the adjacent chain when they are completely separated, the pulling work was defined at the saturation stage (Figure 5). *W*_pull_ obtained for all the systems is shown in Table S3. For the tetramer, the average *W*_pull_ value is 347 kcal/mol, which is lower than 977 kcal/mol of 5OQV but higher than 312 kcal/mol of 2NAO (Table 1). This means that the oligomers are less stable than 5OQV but more stable than 2NAO. However, averaging over two fibril structures, we obtain *W*_pull_ = (977 + 312)/2 = 644.5 kcal/mol, which is higher than 347 kcal//mol of the tetramer. Thus, as in the *F*_max_ case, our data on the pulling work also support the trend reported in experimental work^9^ that the fibril is more stable than oligomers. We do not compare the mechanical stability of the tetramer with 2BEG because this fibril structure was obtained for the truncated version of Aβ_17–42_, but not for the full-length peptide.

### Hydrogen Bonds Plays a Key Role in Stability of Aβ Aggregates

Experimentally, Ruggeri et al.^9^ found that hydrogen bonds (HBs) between β-sheets are the main factor determining the stability of Aβ aggregates. Moreover, during the aggregation process, oligomers possess partial β-sheet conformations.^50^ The number of HBs and β-content of all initial structures were calculated (Table 2). In terms of *F*_max_ and *W*_pull_ (Table 1), the 5OQV fibril is much more stable compared to 2NAO, which is well correlated to the fact that 5OQV has 105 HBs vs 24 HBs of 2NAO. 5OQV also has the β-content (79.6%) higher than 2NAO (38.5%). Thus, in agreement with experiment, the more HBs and β-content, the more stable the fibrils.

**Table 2:** Number of Hydrogen Bonds and β-Content of Initial Structures of Tetramers and Fibrils

Aβ system	number of HBs	β content (%)
cluster 1	22	16.7 ± 2.9
cluster 2	13	10.1 ± 2.3
cluster 3	15	16.1 ± 2.8
cluster 4	20	21.4 ± 3.2
cluster 5	16	28.0 ± 3.5
2NAO	24	38.5 ± 3.1
5OQV	105	79.6 ± 2.1
2BEG	57	77.7 ± 3.7

For tetramers, clusters 1 and 4 have more HBs than clusters 2, 3, and 5 (Table 2). On the other hand, the rupture force and pulling work of models 1 and 4 are larger (Tables 1) than the remaining models, which also confirms the importance of HBs in the stability of Aβ complexes.

### Contribution of Interchain Electrostatic and van der Waals Interactions to Nanomechanical Stability Depends on the Aβ Structures

To understand the role of interchain electrostatic and van der Waals (vdW) interactions, we calculated them in initial structures (Table S4) and their time dependence in SMD simulations (Figure S3). Electrostatic interaction is more important than vdW for 5OQV and 2BEG, but vice versa in the case of 2NAO. In the case of a tetramer, the vdW interaction is more important than electrostatic for clusters 2, 3, and 5, but for clusters 1 and 4, the latter dominates. Because 5OQV is mechanically more stable than 2NAO, and models 1 and 4 have higher breaking force than models 2, 3, and 5 (Table S2), we would expect electrostatic interaction to dominate the vdW interaction in highly stable structures. It would be interesting to test this conclusion on other force fields and water models.

### Mechanical Stability Depends on the Morphology of the Fibril

The 2NAO fibril has a S-bend topology, while 5OQV has a LS-shape (Figure 3), leading to different mechanical stability (Table 1). Thus, we hypothesize that mechanical stability depends on the fibril morphology. To further validate this hypothesis, we performed SMD simulations for 2BEG, which has a U-bend structure. Typical force–extension profiles are shown in Figure 4; *W*_pull_ and *F*_max_ obtained for 10 trajectories are presented in Tables S2 and S3, respectively. The average rupture force (1416 pN) and pulling work (188 kcal/mol) (Table 1) are lower than those of 2NAO and 5OQV, which supports the hypothesis of the dependence of stability on the fibril structure.

### Robustness of Results against Pulling Speeds

To show that our main conclusion about the higher stability of fibrils in comparison with oligomers does not depend on pulling speed, we simulated a tetramer and 2NAO fibril at *v* = 5 nm/ns. From Table S5, for the tetramer we obtained the average pulling work *W*_pull_ ≈ 681 kcal/mol, which is comparable with *W*_pull_ ≈ 691 kcal/mol of 2NAO. However, averaging the results obtained for five clusters of the tetramer (Table S6), we have *F*_max_ ≈ 1624 pN, which is lower than *F*_max_ ≈ 2271 pN of 2NAO. Thus, the lower stability of oligomers compared to that of fibrils does not depend on the pulling speed.

## Coarse-Grained Simulation

### Young Modulus of Aβ Tetramers Is Less than Fibril-like Structures

**Figure 6:**
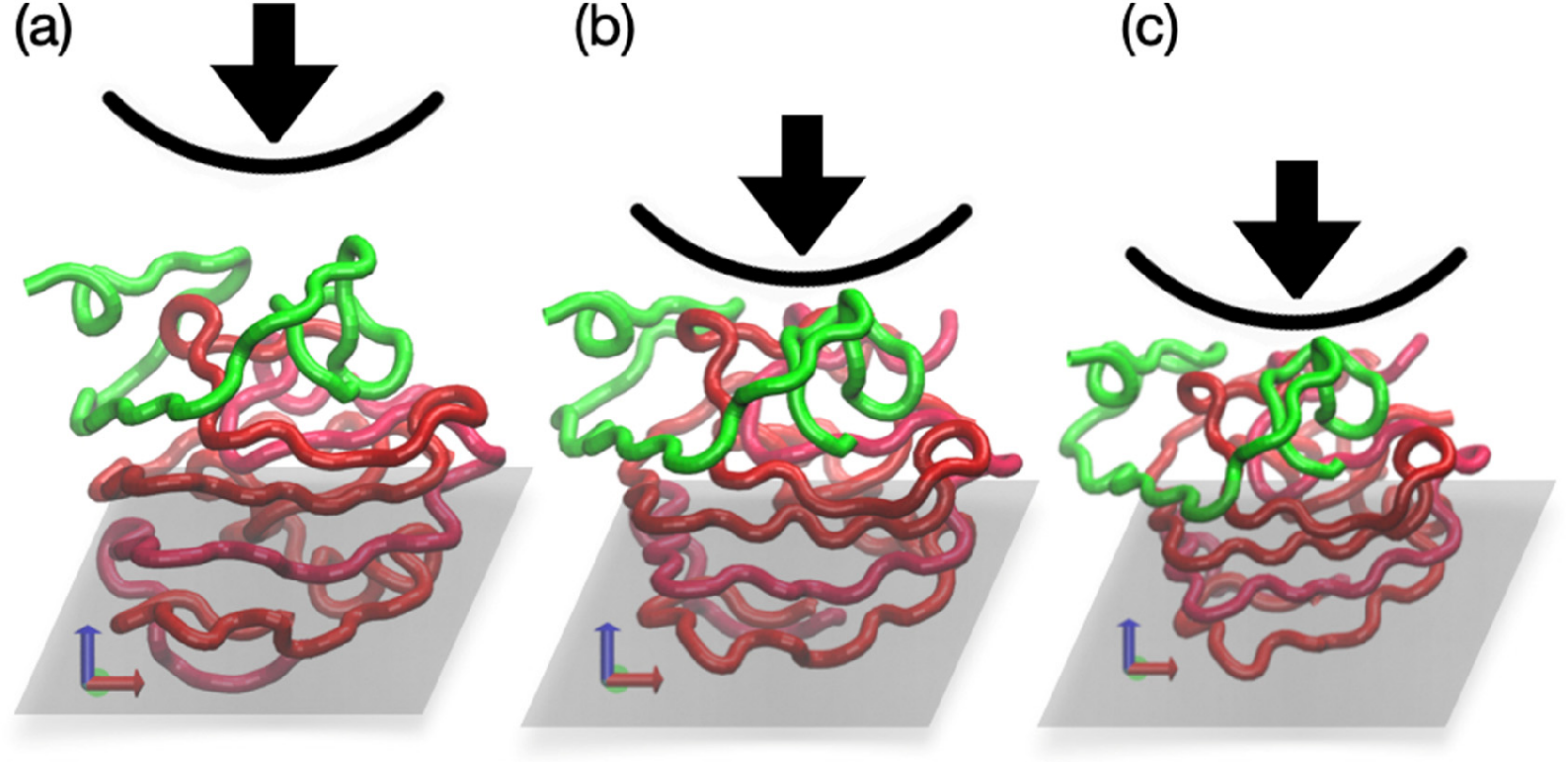
CG nanoindentation of Aβ_42_ tetramers. Here we show the typical deformation process induced by a spherical indenter with *R*_ind_ = 10 nm at different penetration depths (*h*) as follows: (a) *h* = 0 nm (no contact), (b) *h* = 1 nm (moderate deformation), and (c) *h* = 1.8 nm (large deformation). The top protein chain is highlighted in green color to indicate the first chain in the tetramer which is in contact with the indenter. The repulsive plane is also depicted for each snapshot.

**Figure 7:**
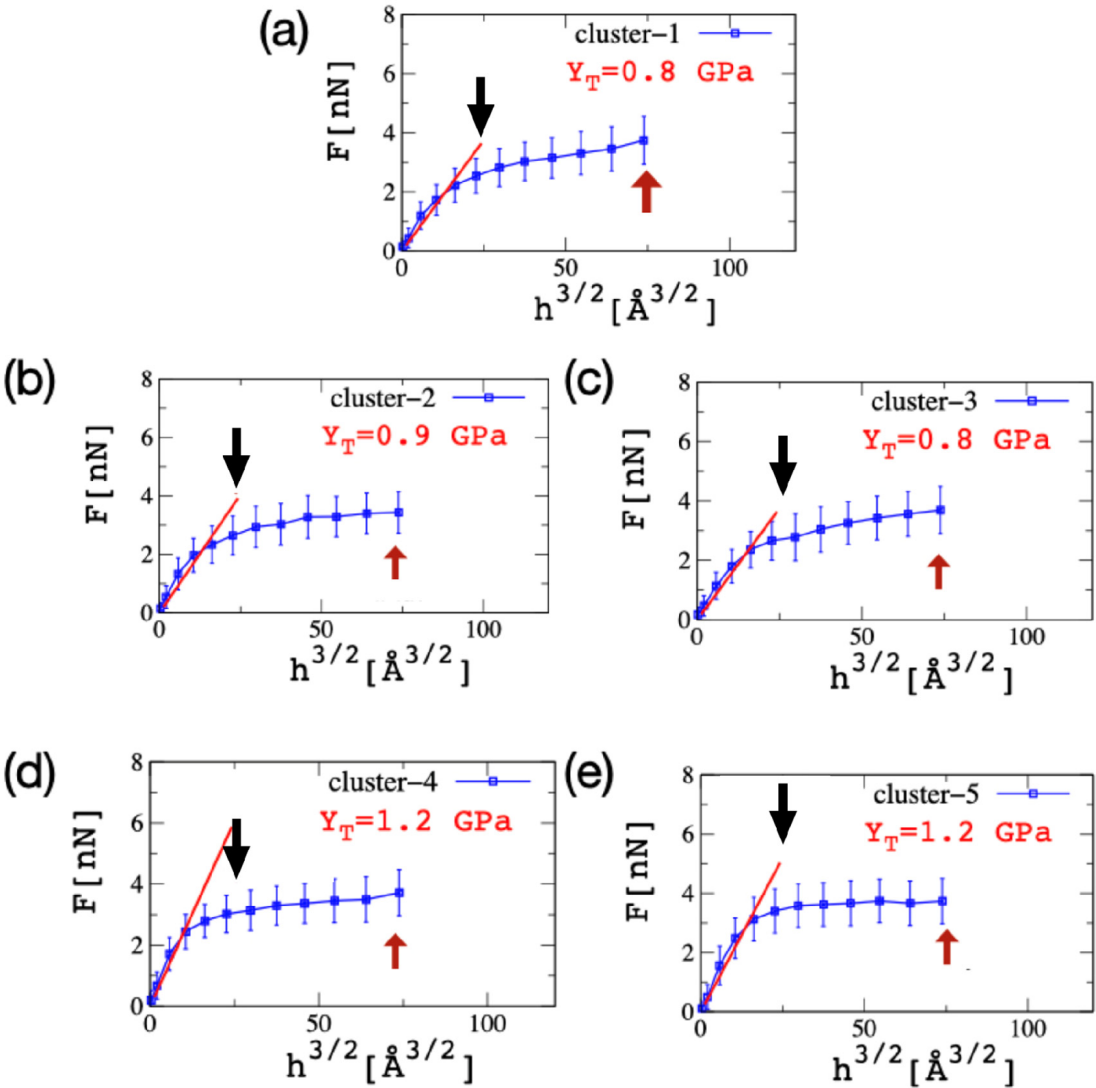
CG nanoindentation profiles of five Aβ tetramer clusters. The Young modulus is presented next to the linear fit and shows a deviation in the range 0.8–1.2 GPa. Vertical arrows show the position of the indenter at 1 and 1.8 nm as depicted in Figure 5.

The computational nanoindentation of Aβ_42_ tetramers is described below. *In vitro* studies^9^ provide evidence of the relative difference in Young modulus (*Y*_T_) between the fibril-like structure and the Aβ_42_ oligomers. Our results consider the size of the spherical indenter (*R*_ind_) equal to 10 nm and the expected force (*F*) to be between few nanonewtons when *h* is about 1 nm. We take *v*_ind_ of 5 × 10^–7^ nm/ps to match the experimental time scale. Figure 6 shows snapshots of the indentation process for an Aβ tetramer. The computational nanoindentation results are reported in Figure 7. Here we show the plot of *F* vs *h*^3/2^, and the linear fit that is used to determine *Y*_T_. We find that the values of *Y*_T_ for Aβ tetramers vary in the range 0.8–1.2 GPa, which is in agreement with AFM studies.^9^ Our previous work^39^ showed a systematic comparison between *in silico* and *in vitro* experiments for the calculation of the *Y*_T_ modulus in Aβ_42_ and Aβ_40_ forming fibril structures. We reported for those systems a *Y*_T_ in the ranges 3–10 and 7–21 GPa for Aβ_42_ and Aβ_40_, respectively. Such a deviation in the Young modulus was associated with the type of amyloid symmetry and the high degree of crystal-like order retained in the CG model.

This result was also validated by *in vitro* experiments for the related Aβ_42_ fibril, and it provides 3.3 GPa.^9^ The experimental value of the fibril-like Aβ_40_ has not yet been reported, but it is expected to fall in the range 2–4 GPa according to other amyloid systems (e.g., assembled from α-synuclein, heptapeptides, insulin, β-lactoglobulin, tau protein, lysozyme, ovalbumin, and bovine serum albumin).^51,52^ Those studies show how softer tends to be the oligomer state in comparison to the fibril-like state. According to our *in silico* studies here, we found that the *Y*_T_ drops by a factor of 10 in tetramers compared to the fibril system. This result indicates the loss of cooperative effect due to weak and unoriented native contacts in the Aβ tetramers state compared to fibril-like state. Overall, structure-based CG and SMD simulations provide similar results for the mechanical stability of Aβ aggregates.

## Conclusions

Our work shows the important role of mechanical stability in Aβ systems, from the initial tetramer cluster formation to more stable fibril-like structures. Further studies can shed light onto the gain of the nanomechanical stability as a function of time in adhesion of proteins and their aggregation pathway. In addition, CG simulation based on the Cα backbone and the mapping all native interactions for each Aβ tetramer cluster show consistent results with all-atom MD. The indentation process was performed by a spherical hard object with a radius of curvature equal to 10 nm. Once it was in contact with the Aβ tetramer, the response of the system while pressing it against the direction of maximal HB deformation, a linear fitting was sufficient to show small variations in the Young modulus in the range 0.8–1.2 GPa. Such deviations are in agreement with experimental results.^9^

Recently, the MARTINI force field has drawn attention to the calculation of mechanical properties of biomolecules. Fontana and Gelain^53^ were able to assess the Young modulus from stretching simulations. So far, to our knowledge, the MARTINI force field has not been used to perform nanoindentation, so it would be interesting to use it to compare the stability of amyloid oligomers and fibrils.

## Supporting Information Available

Figure S1: time dependence of the root-mean-square deviation (RMSD) of 5OQV at pH = 2 and pH = 7; Figure S2: contact map which shows only the contacts that have existed in the initial structure but are broken at the rupture force; Figure S3: time dependence of the interchain interaction energy, obtained in SMD simulation; Table S1: numbers of interchain contacts between the pulled chain and the other chains of the initial structures and structures at *F*_max_; Table S2: the rupture force *F*_max_ of Aβ tetramer and fibril-like structures for 20 trajectories; Table S3: the pulling work of the Aβ tetramer and fibril-like structures for 20 trajectories; Table S4: interchain interaction energy of initial structures; Table S5: the pulling work of the Aβ tetramer and 2NAO was obtained for pulling speed *v* = 5 nm/ns and 10 trajectories; Table S6: *F*_max_ of the Aβ tetramer and fibril-like structure 2NAO; results obtained for *v* = 5 nm/ns and SMD 10 trajectories (PDF)
