# Supplementary material for: Nanomechanical Stability of Aβ Tetramers and Fibril-like Structures: Molecular Dynamics Simulations
Source: J Phys Chem B. 2021 Jul 12;125(28):7628–37. doi: 10.1021/acs.jpcb.1c02322 (PMC8389904; doi:10.1021/acs.jpcb.1c02322)
Supplement: Supplementary file 1 — jp1c02322_si_001.pdf [file jp1c02322_si_001.pdf]

SUPPORTING INFORMATION

Nanomechanical Stability of Aβ Tetramers and Fibril-like Structures: Molecular Dynamics Simulations

Adolfo B. Poma [a,g,*,†], Tran Thi Minh Thu[b,c,e*], Lam Tang Minh Tri[c,e], Hoang Linh Nguyen[b,d,e], and Mai Suan Li[f,†]

a. Institute of Fundamental Technological Research, Polish Academy of Sciences, Pawińskiego 5B, 02-106, Warsaw

b. Institute for Computational Science and Technology, SBI Building, Quang Trung Software City, Tan Chanh Hiep Ward, District 12, Ho Chi Minh City, Viet Nam

c. Faculty of Materials Science and Technology, Ho Chi Minh City University of Science - VNUHCM, 227 Nguyen Van Cu Street, District 5, Ho Chi Minh City, Viet Nam

d. Ho Chi Minh City University of Technology (HCMUT), Ho Chi Minh City 700000, Vietnam

e. Vietnam National University, Ho Chi Minh City 700000, Vietnam

f. Institute of Physics, Polish Academy of Sciences, Al. Lotników 32/46, 02-668 Warsaw, Poland

g. International Center for Research on Innovative Biobased Materials (ICRI-BioM)—International Research Agenda, Lodz University of Technology, Żeromskiego 116, 90-924 Lodz, Poland

* These authors contributed equally

† Corresponding author: Mai Suan Li (masli@ifpan.edu.pl), Adolfo B. Poma (apoma@ippt.pan.pl).

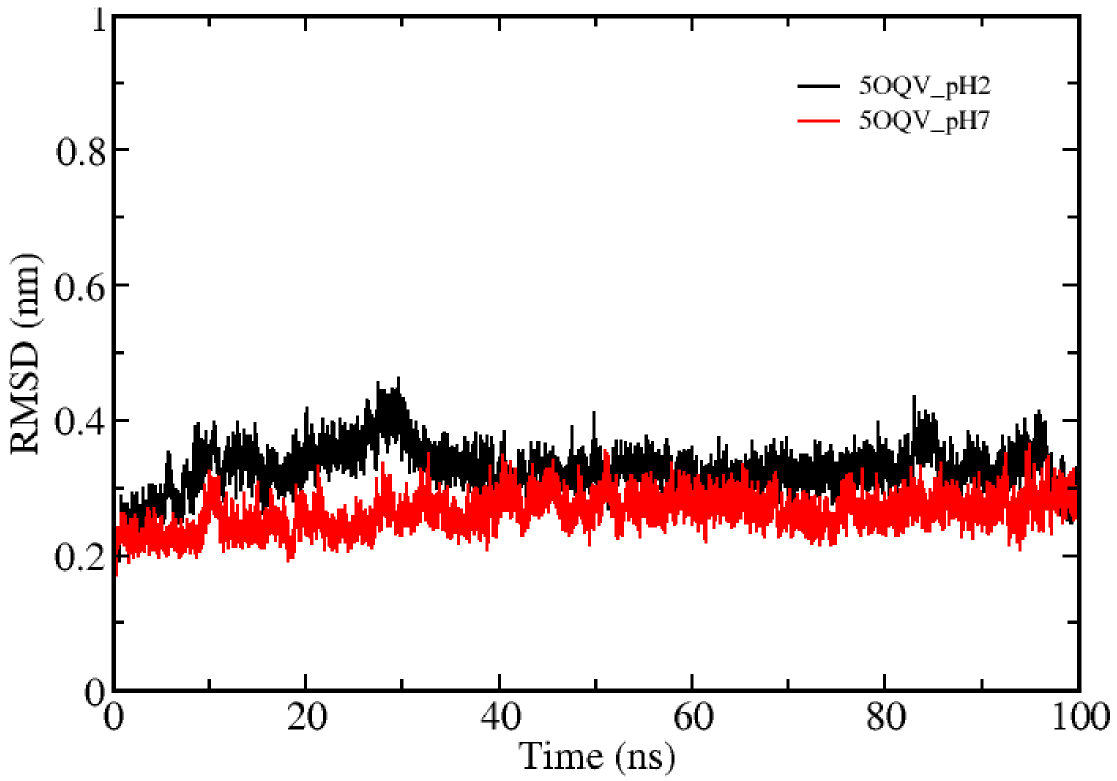

Figure S1. Time dependence of the root mean square deviation (RMSD) of 5OQV at pH=2 and pH=7. MD simulation was conducted at 300K using TIP3P water model and CHARMM36m force field.

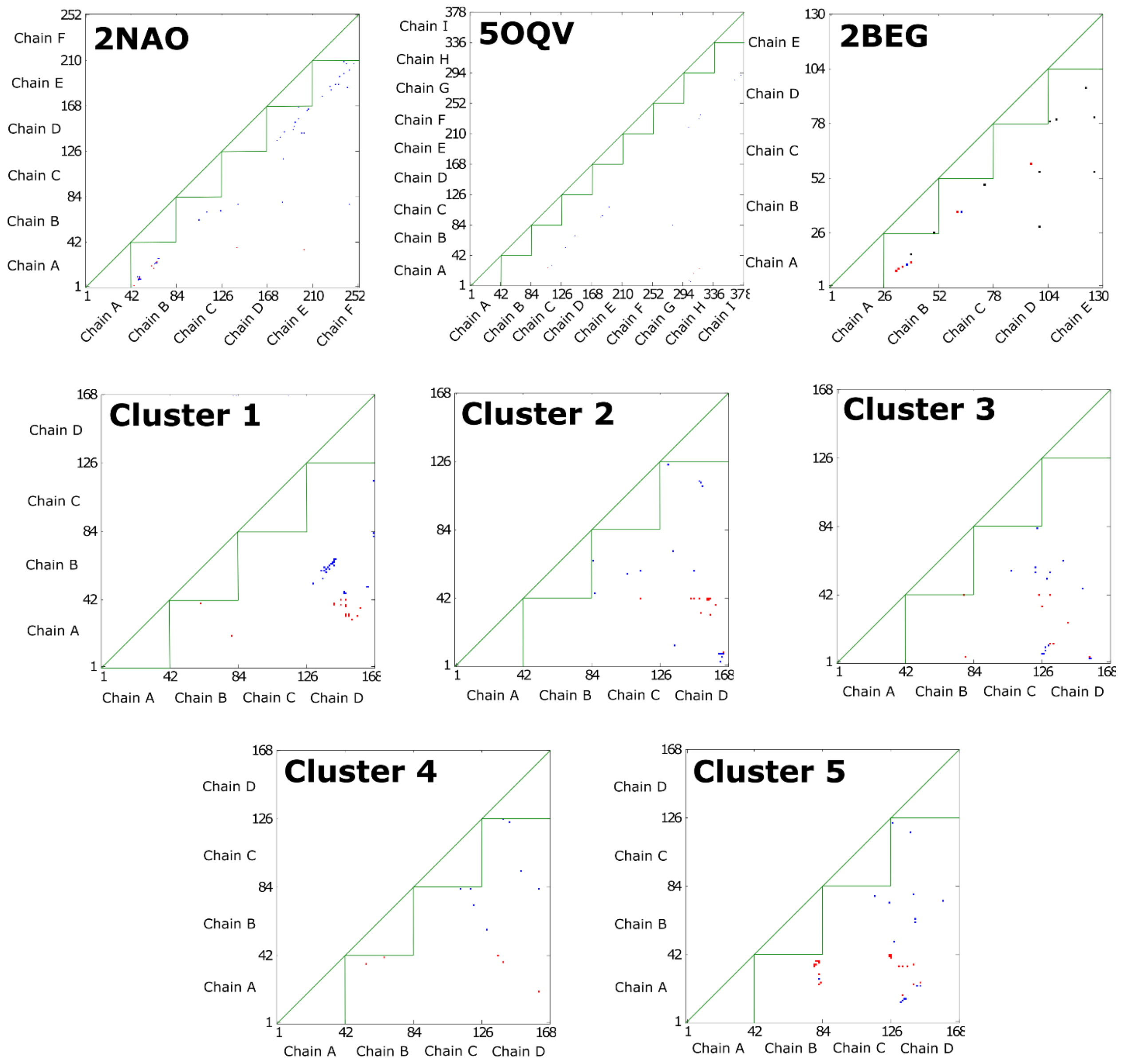

Figure S2. Contact map which shows only the contacts that have existed in the initial structure but are broken at the rupture force. Black, red, and blue refer to hydrophobic-hydrophobic, hydrophobic-hydrophilic and hydrophilic-hydrophilic contacts, respectively

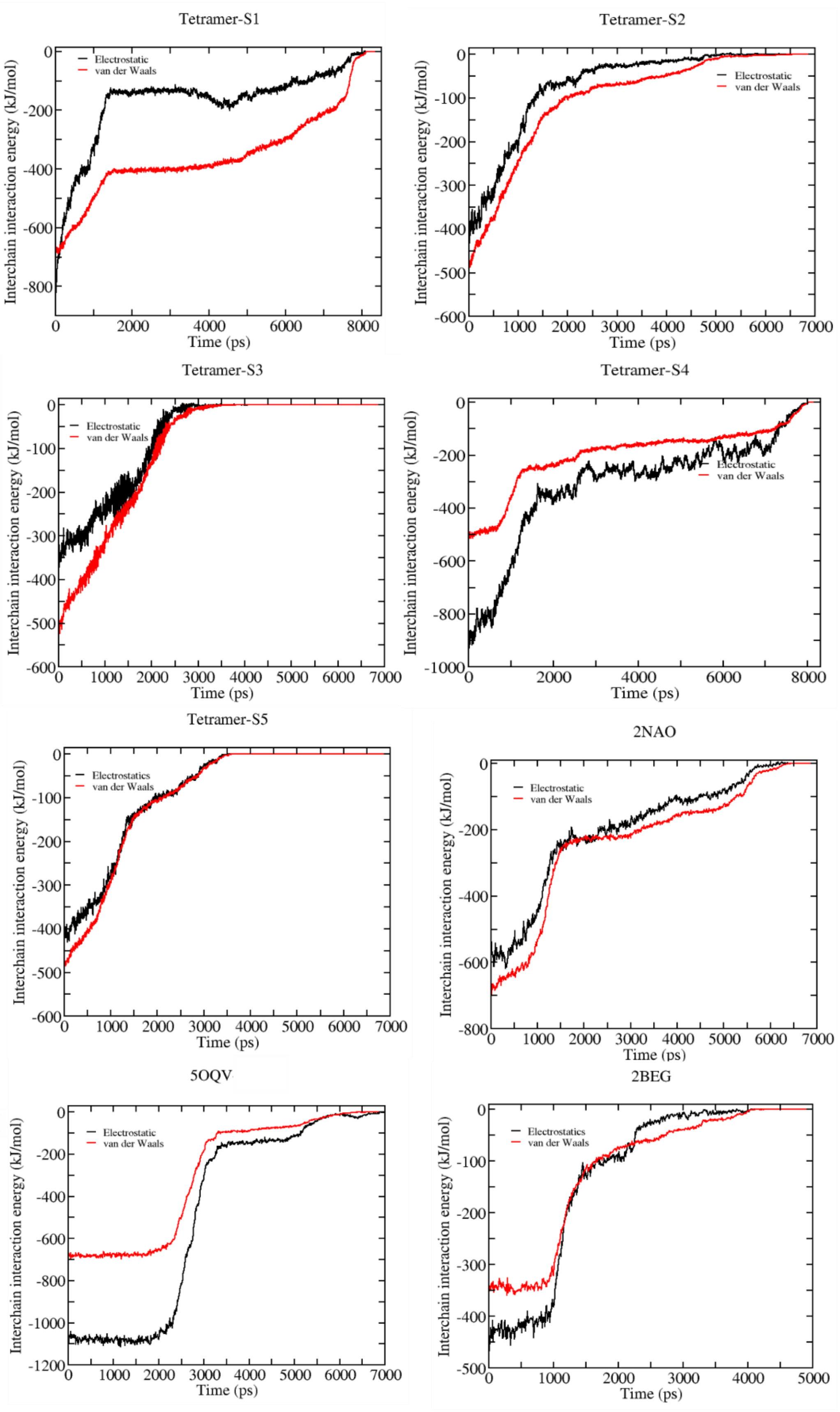

Figure S3. Time dependence of the interchain interaction energy, obtained in SMD simulation. Black and red denote the electrostatic and vdW interaction, respectively.

Table S1. Numbers of interchain contacts between the pulled chain and the other chains of the initial structures and structures at F_{max}. A contact is formed if the distance between the centers of mass of the side chains exceeds 0.65 nm. Hydrophobic residues are glycine (Gly), alanine (Ala), valine (Val), leucine (Leu), isoleucine (Ile), proline (Pro), phenylalanine (Phe), methionine (Met), and tryptophan (Trp). The remaining residues are hydrophilic.

System	configuration	Hydrophobic-hydrophobic	Hydrophobic-hydrophilic	Hydrophilic-hydrophilic
2NAO	Initial	40	11	19
	at Fmax	28.95	4.85	6.25
5OQV	Initial	44	6	22
	at Fmax	38.70	5	16.30
2BEG	Initial	33	6	6
	at Fmax	18.7	1.70	2.45
Cluster 1	Initial	55	14	5
	at Fmax	33.25	2.85	0.00
Cluster 2	Initial	39	16	3
	Fmax	20.40	7.80	1.80
Cluster 3	Initial	18	15	8
	at Fmax	13.40	7.80	4.15
Cluster 4	Initial	28	25	4
	at Fmax	19.84	19.10	2.53
Cluster 5	Initial	20	18	7
	at Fmax	4.22	11.25	5.45

Table S2. The rupture force F_{max} of Aβ tetramer and fibril-like structures for 20 trajectories **and** pulling rate v = 1nm/ns.

	F_{max} (pN)							
	Cluster 1	Cluster 2	Cluster 3	Cluster 4	Cluster 5	2NAO	5OQV	2BEG
traj1	1808	568	895	1184	885	1665	3357	1339
traj2	2183	828	922	1083	724	1605	4118	2017
traj3	1677	895	781	1358	936	1649	3806	1518
traj4	2042	657	785	996	1067	1557	3828	1482
traj5	2092	565	800	1103	808	1708	2847	1726
traj6	1901	567	672	1567	816	1722	4386	1805
traj7	2640	623	833	1218	852	1145	4704	1472
traj8	1935	738	761	1186	1125	1623	4366	1441
traj9	2001	786	740	1624	782	1380	4487	1446
traj10	2005	700	777	1000	1045	1300	4896	1475
traj11	2251	626	696	997	904	1650	3992	1233
traj12	2054	836	943	1020	1059	1514	3743	1334
traj13	2082	572	987	1215	882	1397	4139	1222
traj14	1942	1059	644	1384	1178	1724	4281	1321
traj15	1989	568	1025	1570	1301	1396	5265	1203
traj16	1910	936	803	1114	1243	1364	4656	1212
traj17	2115	653	818	1320	1013	1358	4092	1296
traj18	2354	594	781	1416	829	1613	4846	1282
traj19	2075	675	776	941	1380	1695	4391	1306
traj20	2242	615	891	1222	800	1607	3802	1184
Average F_{max} (pN)	2065 ±207	703 ±143	817 ±101	1226 ±207	981 ±188	1534 ±166	4200 ±560	1416 ±219

Table S3. The pulling work of the Aβ tetramer and fibril-like structures for 20 trajectories. It was calculated using Eq. (1), pulling rate v = 1nm/ns

	W_{pull} (kcal/mol)							
	Cluster 1	Cluster 2	Cluster 3	Cluster 4	Cluster 5	2NAO	5OQV	2BEG
traj1	822	95	188	424	211	249	761	243
traj2	885	104	185	401	155	308	943	270
traj3	759	130	148	420	136	308	823	167
traj4	747	155	148	325	141	306	861	191
traj5	928	123	168	348	143	356	643	229
traj6	553	95	149	479	99	337	952	252
traj7	1004	156	187	431	171	297	1189	213
traj8	952	126	144	327	211	348	1030	214
traj9	881	126	150	476	156	305	1036	161
traj10	985	122	138	299	180	331	1194	185
traj11	922	115	146	370	141	317	906	137
traj12	865	151	195	346	143	277	824	189
traj13	896	137	190	445	170	307	935	129
traj14	843	161	136	332	249	350	1067	155
traj15	898	116	148	499	210	341	1337	193
traj16	851	198	156	272	209	311	1125	153
traj17	904	106	145	371	151	253	891	219
traj18	994	133	160	419	176	329	1166	154
traj19	965	169	153	350	248	296	983	173
traj20	987	143	170	379	162	330	867	124
Average W_{pull}	882±106	133±26	160±19	386±63	173±39	313±29	977±169	188±42

Table S4. Interchain interaction energy of initial structures

System	Electrostatic energy (kJ/mol)	vdW energy (kJ/mol)
2NAO	-533.4	-707.2
5OQV	-1137.1	-656.3
2BEG	-493.0	-337.5
Cluster 1	-776.7	-705.6
Cluster 2	-480.7	-562.5
Cluster 3	-438.2	-527.4
Cluster 4	-949.8	-513.5
Cluster 5	-417.1	-523.3

Table S5. The pulling work of the Aβ tetramer and 2NAO was obtained for pulling speed v = 5nm/ns and 10 trajectories.

	W_{pull} (kcal/mol)					
	Cluster 1	Cluster 2	Cluster 3	Cluster 4	Cluster 5	2NAO
traj1	1488	435	458	965	430	701
traj2	1376	307	409	765	426	586
traj3	1509	362	398	761	324	666
traj4	1441	469	406	853	428	901
traj5	1412	376	433	829	237	661
traj6	1401	299	450	787	374	622
traj7	1407	358	444	752	328	775
traj8	1409	376	407	617	415	691
traj9	1404	347	447	846	458	598
traj10	1409	322	425	865	435	707
Average W_{pull}	1426±42	365±54	428±22	804±92	385±69	691±93

Table S6. F_{max} of the Aβ tetramer and fibril-like structure 2NAO. The results were obtained for v = 5 nm/ns and SMD 10 trajectories.

	F_{max} (pN)					
	Cluster 1	Cluster 2	Cluster 3	Cluster 4	Cluster 5	2NAO
traj1	2732.8	1507.8	1338.1	2078.3	1451.0	2247.3
traj2	1987.2	1149.7	1378.3	1591.1	1222.5	2117.8
traj3	2260.9	1093.8	1500.5	2022.1	1345.8	2084.8
traj4	2058.0	1381.4	1407.7	2022.8	1323.3	3149.7
traj5	2062.6	1188.5	1370.6	1872.4	1307.2	2249.3
traj6	2220.7	969.3	1284.0	1721.5	1724.9	1885.5
traj7	2134.2	1069.4	1653.9	1629.6	1257.4	2383.3
traj8	2293.2	1240.9	1319.8	1583.7	1400.4	2418.8
traj9	2311.9	1193.1	1469.9	1927.8	1468.1	1893.4
traj10	2603.3	1195.6	1535.5	2072.3	1274.4	2276.2
Average F_{max}	2266.5 ± 239.5	1198.9 ± 154.1	1425.8 ± 113.6	1852.2 ± 202.9	1377.5 ± 146.4	2270.6 ± 358.5
